# Supplementary material for: Development of a computer-aided design software for the quantitative evaluation of aesthetic damage
Source: PLoS One. 2019 Dec 18;14(12):e0226322. doi: 10.1371/journal.pone.0226322 (PMC6919621; doi:10.1371/journal.pone.0226322)
Supplement: S1 Code site — 1. (ZIP) [file pone.0226322.s001.zip › [EESC_jr][Projeto]Relatorio_07_21/reports/templates_zip/gentelella-master/vendors/echarts/test/treemap-obama.html]

示例模式  
(Mode)

面积代表“2012年预算额” (Area represents '2012 Amount)  

面积代表“2012年预算额”，明暗代表“2011年相比的增长率”  
     (Area represents '2012 Amount' and color-alpha represents 'Change from 2011')  

面积代表“2011年预算额” (Area represents '2011 Amount')
